# 1088. A Whole-Body Quantitative System Pharmacology Physiologically-Based Pharmacokinetic (QSP/PBPK) Model to Support Dose Selection of ADG20: an Extended Half-Life Monoclonal Antibody Being Developed for the Treatment of Coronavirus Disease (COVID-19)

**DOI:** 10.1093/ofid/ofab466.1282

**Published:** 2021-12-04

**Authors:** Evan D Tarbell, Scott A Van Wart, Dhaval K Shah, Laura M Walker, Andrew Santulli, Lynn E Connolly, Donald E Mager, Ashley N Brown, Paul G Ambrose

**Affiliations:** 1 Enhanced Pharmacodynamics LLC, Buffalo, New York; 2 University at Buffalo School of Pharmacy and Pharmaceutical Sciences, Buffalo, New York; 3 Adimab LLC, Lebanon, New Hampshire; 4 Adagio Therapeutics, Inc., Waltham, Massachusetts; 5 University of Florida, College of Medicine, Orlando, Florida

## Abstract

**Background:**

ADG20 is a fully human IgG1 monoclonal antibody engineered to have potent and broad neutralization against severe acute respiratory syndrome coronavirus 2 (SARS-CoV-2) and other SARS-like CoVs with pandemic potential and an extended half-life. ADG20 is administered intramuscularly (IM). A QSP/PBPK model was constructed to support dose selection for a Phase 2/3 trial of ambulatory patients with mild to moderate COVID-19 (STAMP: NCT04805671).

**Methods:**

A QSP/PBPK model was used to simulate receptor occupancy (RO) and drug exposure in the upper airway (nasopharyngeal/oropharyngeal epithelial lining fluid [ELF] compartment). RO was linked to an existing viral dynamic model to enable the prediction of the natural time course of viral load and the effect of ADG20 on viral clearance and infectivity rate. RO was calculated using: 1) in vitro ADG20–SARS-CoV-2 binding kinetics (association rate constant (k_on_) of 1.52E+06 M^-1^•s^1^ and dissociation rate constant (k_off_) of 2.81E-04 s^-1^ from a Biacore assay; 2) time course of ADG20 concentrations in ELF; and 3) time course of viral load following ADG20 administration. Molar SARS-CoV-2 viral binding site capacity was calculated assuming 40 spike proteins per virion, 3 binding sites per spike, and an initial viral load of log 10^7^ copies/mL for all patients. The QSP/PBPK model and a 2018 CDC reference body weight distribution (45–150 kg) were used to simulate 1000 concentration-time profiles for a range of candidate ADG20 regimens. ADG20 regimens were evaluated against 2 criteria: 1) ability to attain near complete ( >90%), and durable (28-day) SARS-CoV-2 RO in the ELF; and 2) ability to maintain ELF ADG20 concentrations relative to a concentration (0.5 mg/L) associated with 100% viral growth suppression in an in vitro post-infection assay.

**Results:**

A single 300 mg IM ADG20 dose met the dose selection criteria in terms of RO (Figure A) and viral growth suppression (Figure B).

**Conclusion:**

These data support the evaluation of an ADG20 300 mg IM dose for the treatment of mild to moderate COVID-19. ADG20 is forecasted to attain near complete ( >90%) SARS-CoV-2 RO in the ELF and maintain ELF ADG20 concentrations above that associated with 100% viral growth suppression in vitro.

Figure. QSP/PBPK model forecast of ADG20 300 mg IM in adults

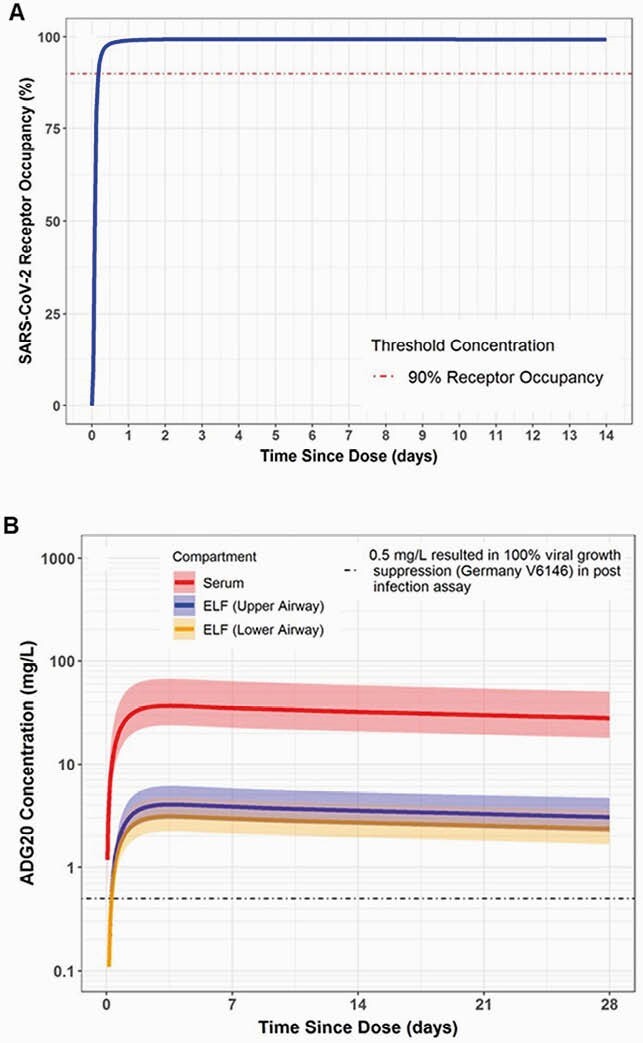

(A) Predicted RO expressed as percent occupancy with the dotted line representing the threshold for 90% RO. (B) Predicted median concentration of ADG20 relative to a concentration (0.5 mg/L) associated with 100% viral growth suppression as indicated by the dotted line; the shaded area represents the 90% prediction interval.

**Disclosures:**

**Evan D. Tarbell, PhD**, **Adagio Therapeutics, Inc.** (Independent Contractor) **Scott A. Van Wart, PhD**, **Adagio Therapeutics, Inc.** (Independent Contractor) **Laura M. Walker, PhD**, **Adagio Therapeutics, Inc.** (Other Financial or Material Support, Laura M. Walker is an inventor on a patent application submitted by Adagio Therapeutics, Inc., describing the engineered SARS-CoV-2 antibody.) **Andrew Santulli, PhD**, **Adagio Therapeutics, Inc.** (Independent Contractor) **Lynn E. Connolly, MD, PhD**, **Adagio Therapeutics, Inc.** (Employee) **Donald E Mager, PharmD, PhD**, **Adagio Therapeutics, Inc.** (Independent Contractor) **Paul G. Ambrose, PharmD**, **Adagio Therapeutics, Inc.** (Employee)

